# Ethosomes for Curcumin and Piperine Cutaneous Delivery to Prevent Environmental-Stressor-Induced Skin Damage

**DOI:** 10.3390/antiox13010091

**Published:** 2024-01-11

**Authors:** Francesca Ferrara, Agnese Bondi, Walter Pula, Catia Contado, Anna Baldisserotto, Stefano Manfredini, Paola Boldrini, Maddalena Sguizzato, Leda Montesi, Mascia Benedusi, Giuseppe Valacchi, Elisabetta Esposito

**Affiliations:** 1 Department of Chemical, Pharmaceutical and Agricultural Sciences, University of Ferrara, 44121 Ferrara, Italy; frrfnc3@unife.it (F.F.); agnese.bondi@unife.it (A.B.); walter.pula@unife.it (W.P.); catia.contado@unife.it (C.C.); sgzmdl@unife.it (M.S.); 2 Department of Life Sciences and Biotechnology, University of Ferrara, 44121 Ferrara, Italy; bldnna@unife.it (A.B.); stefano.manfredini@unife.it (S.M.); 3 Center of Electron Microscopy, University of Ferrara, 44121 Ferrara, Italy; paola.boldrinicme@unife.it; 4 Cosmetology Center, University of Ferrara, 44121 Ferrara, Italy; leda.montesi@unife.it; 5 Department of Neurosciences and Rehabilitation, University of Ferrara, 44121 Ferrara, Italy; mascia.benedusi@unife.it; 6 Animal Science Department, NC Research Campus, Plants for Human Health Institute, NC State University, Kannapolis, NC 28081, USA; 7 Department of Food and Nutrition, Kyung Hee University, Seoul 26723, Republic of Korea

**Keywords:** ethosomes (ETs), curcumin (CUR), piperin (PIP), skin, environmental stress

## Abstract

Diesel particulate matter is one of the most dangerous environmental stressors affecting human health. Many plant-derived compounds with antioxidant and anti-inflammatory properties have been proposed to protect the skin from pollution damage. Curcumin (CUR) has a plethora of pharmacological activities, including anticancer, antimicrobial, anti-inflammatory and antioxidant. However, it has low bioavailability due to its difficult absorption and rapid metabolism and elimination. CUR encapsulation in nanotechnological systems and its combination with biopotentiators such as piperine (PIP) can improve its pharmacokinetics, stability and activity. In this study, ethosomes (ETs) were investigated for CUR and PIP delivery to protect the skin from damage induced by diesel particulate matter. ETs were produced by different strategies and characterized for their size distribution by photon correlation spectroscopy, for their morphology by transmission electron microscopy, and for their drug encapsulation efficiency by high-performance liquid chromatography. Franz cells enabled us to evaluate in vitro the drug diffusion from ETs. The results highlighted that ETs can promote the skin permeation of curcumin. The studies carried out on their antioxidant activity demonstrated an increase in the antioxidant power of CUR using a combination of CUR and PIP separately loaded in ETs, suggesting their possible application for the prevention of skin damage due to exogenous stressors. Ex vivo studies on human skin explants have shown the suitability of drug-loaded ETs to prevent the structural damage to the skin induced by diesel engine exhaust exposure.

## 1. Introduction

Environmental pollution is a worldwide recognized serious concern, affecting human health and the ecosystem. Indeed, many harmful solids, liquids and gases are hugely introduced into the environment due to modern unprecedented urbanization and industrialization, resulting in deleterious effects, involving cardiovascular, respiratory, reproductive and skin damage [1,2,3]. Among pollutants, diesel engine exhaust (DEE) is a ccomplex mixture of gases and particulate matter, responsible for a plethora of systemic ccpathologies as well as skin conditions spanning from atopic dermatitis, eczema and ageing to melanoma [4,5]. Indeed, since the skin is an outer body organ, it represents, on the one hand, the first barrier against environmental factors and, on the other, the common route by which chemicals can enter the body. Nonetheless, since the skin’s defensive potential in some cases can be insufficient to counteract the effect of environmental stressor exposure, a therapeutic and/or protective approach is desirable [6,7].

Many phytocompounds, such as CURand PIP, possess therapeutic properties due to their pleiotropic potential [8,9]. CUR, a polyphenol derived from the rhizome of the East Indian plant *Curcuma longa*, besides being employed as a food additive, is recognized for its antitumor, anti-inflammatory, antioxidant, antiangiogenic, antiviral, antibacterial, antifungal and chemoprotective effects [10,11,12]. In addition, its potential in the treatment of many skin conditions, including acne, alopecia, atopic dermatitis, facial photoaging, oral lichen planus, pruritus, psoriasis, radiodermatitis and vitiligo, has been demonstrated [13].

Notwithstanding CUR’s tolerability even at high doses, it is scarcely bioavailable, mainly because of its poor absorption, rapid metabolism and rapid elimination, limiting its therapeutic potential [14]. To increase its absorption, CUR has been investigated in association with PIP, acting as a natural enhancer [15,16,17,18]. The alkaloid PIP, mainly isolated from the seeds of *Piper nigrum*, is a natural dietary supplement with a distinct pungent flavor. PIP possesses therapeutic potential due to its antipyretic, antifungal, antioxidant, antimutagenic, anticancer, antiepileptic, anti-inflammatory, antioxidant and immunomodulatory actions [9,19,20]. In addition, PIP is remarkably studied for its role as a biopotentiator, being able to increase the bioavailability and biological effectiveness of the drugs with which it is associated, inhibiting drug efflux transporters and modulating drug resistance pathways [17,21]. Recently, the synergistic effects of CUR with some phytochemicals, such as PIP, have been found, enabling CUR efficacy to be improved in many pathologies as well as in cancer treatment, suggesting the possibility of administering the two drugs in combination [15].

Regarding skin application, PIP has demonstrated its suitability (i) in the treatment of inflammatory skin diseases such as atopic dermatitis and psoriasis [22] and (ii) in enhancing the skin permeation ability of CUR in the form of a composite double-layer CUR/PIP delivery system [23]. Despite their pharmacological potential, both CUR and PIP are characterized by scarce solubility and chemical instability, restricting their therapeutic use [16]. To overcome these drawbacks, nanotechnology approaches have been undertaken aimed at solubilizing, protecting and delivering the drugs through different routes through nanoencapsulation. For instance, the co-loading of CUR and PIP in human serum albumin nanoparticles was investigated against breast cancer cells [24], while recently, a self-nanoemulsifying drug delivery system demonstrated the therapeutic efficiency of CUR and PIP in an animal model of Alzheimer’s disease [25]. Remarkably, the drugs have been individually loaded in monoolein aqueous dispersions, nanoemulsions and polymeric and lipid nanoparticles, as well as in vesicular systems, including liposomes and ETs [26,27,28,29,30,31]. Nevertheless, to the best of our knowledge, the effect of the topical administration of nanoencapsulated CUR in combination with nanoencapsulated PIP has not been investigated yet.

ETs are vesicular systems mainly composed of phosphatidylcholine (PC), water and ethanol (20–45% *v*/*v*) and were first proposed by the Touitou research group [32]. The self-assembling of PC in water in the presence of ethanol results in the formation of double-layered multilamellar vesicles with a mean diameter of around 150–200 nm. With respect to well-known liposomes, ETs are characterized by longer stability, higher softness, and an improved ability to solubilize lipophilic drugs [33,34]. Indeed, in this kind of vesicular system, the combination of PC and ethanol results in a penetration enhancer effect, probably due to a partial disorganization, and in the opening of pores in the *stratum corneum* barrier [35]. In a recent transmission electron microscopy study, we demonstrated the capability of intact ETs to pass through the human skin and to reach deeper skin strata such as the dermis [36]. In this respect, ETs can be thought of as transdermal delivery systems, being able to promote the permeability of entrapped drugs through different skin strata. The ET simple and low-energy-consuming production protocol, together with the biocompatibility of the excipients, make these systems particularly attractive for dermatological applications, as demonstrated by the increasingly growing number of related research articles [37,38,39].

Thus, the aim of the present study is to evaluate the capability of CUR and PIP loaded in ETs to prevent skin damage induced by environmental pollution. Particularly, both CUR and PIP were loaded separately in ETs using two different methodologies. After the selection of the production procedure, the antioxidative effect of the drug-loaded ETs, as well as the drug release and permeability, were studied in vitro. Their efficacy against cutaneous oxidative and structural damage was investigated on skin explants pre-treated with ETs and exposed to DEE, selected as one of the most toxic air pollutants. At last, a patch test was performed on human volunteers to investigate their possible irritation effect under cutaneous administration.

## 2. Materials and Methods

### 2.1. Materials

Curcumin ((E,E)-1,7-bis(4-Hydroxy-3-methoxyphenyl)-1,6-heptadiene-3,5-dione, purity grade 94%, CUR) and piperine (1-piperoylpiperidine, purity grade 97%, PIP) were purchased from Merck Life Science S.r.l. (Milan, Italy). The soybean lecithin (PC) (92% phosphatidylcholine) was Epikuron 200 from Lucas Meyer (Hamburg, Germany). Polytetrafluoroethylene (PTFE, Whatman^®^, Seoul, Republic of Korea) (pore size 200 nm) and STRAT-M^®^ membranes were purchased from Merck Life Science S.r.l. (Milan, Italy). Solvents were of an HPLC grade, and all other chemicals were of an analytical grade.

### 2.2. Preparation of Ethosomes

ETs were alternatively prepared by a bulk cold method or by a microfluidic approach.

#### 2.2.1. Bulk Approach

ETs were prepared in bulk by a previously reported cold method based on the dropwise addition of bidistilled water to a PC–ethanol solution [35]. PC was previously solubilized in ethanol (30 mg/mL) under magnetic stirring (IKA RCT basic, IKA^®^-Werke GmbH and Co., KG, Staufen, Germany). Afterwards, water was slowly added to the PC solution kept under magnetic stirring (750 rpm) for 30 min at 22–25 °C up to a final 70:30 (*v*/*v*) water/ethanol ratio. To prepare CUR- and PIP-loaded ETs, the drug was solubilized in the PC–ethanol solution before the addition of water, obtaining a final CUR and PIP concentration of 0.25 mg/mL. In the case of drug-loaded formulations, the preparation was performed in the dark.

#### 2.2.2. Microfluidic Approach

In the case of the microfluidic approach, ETs were prepared using a cross-junction microfluidic chip based on a Large Droplet Junction Chip (Dolomite, Alfatest, Rome, Italy); the hydrophilic quartz channel is 100 µm in depth, mounted on a chip interface, H, equipped with two 4-way linear connectors.

The PC–ethanol solution (30 mg/mL) was employed as the inner lipid phase (LP), and Milli-Q water was employed as the aqueous outer phases (APs). The flow rates of the APs and LP (respectively, F_AP_ and F_LP_) were regulated using two syringe pumps (IPS-14 syringe series, Inovenso Inc., İstanbul, Turkey). An optical microscope (Leica DM LS2, Leica Microsystems Srl, Buccinasco (MI), Italy) was employed for control. The flow rate ratio (FRR) between F_LP_ and F_AP_ was 2:1 *v*/*v*, and the total flow rates (TFRs) tested were 720, 1440, 2160, 3600 and 5400 µL h^−1^.

After the stabilization of the focused stream, 3 mL of each sample was collected into glass vials and maintained at 4 °C; all samples were prepared at 25 °C.

### 2.3. Transmission Electron Microscopy (TEM)

For the TEM analyses, the ET samples were negatively stained by depositing a sample drop on a TEM grid covered with a formvar film. The excess drop was removed after 1 min from the grid with filter paper to keep a light veil of the sample on the supporting substrate. A drop of 2% phosphotungstic acid was placed on the grid for 1 min and then removed with filter paper to surround the nanosystems deposited on the grid and adhere to their surface. Then, the grid was observed with a TALOS L120C G2 Transmission Electron Microscope (Thermo Fisher Scientific, Eindhoven, The Nederlands).

### 2.4. Photon Correlation Spectroscopy (PCS)

Nanovesicle dimensions and polydispersity indices were ascertained using a Zetasizer Nano S90 (Malvern Instruments, Worcester, UK) equipped with a helium–neon laser of 5 mW and 633 nm emission. The environmental conditions for the measurements were set at ambient temperature (25 °C), with a detection angle of 90°, and the minimum duration of a measurement was fixed at 180 s. A dilution ratio of 1:10 (*v*/*v*) with bidistilled water was standardized for the samples. The analysis of the gathered data was performed utilizing the cumulant analysis technique [39]. These measurements were replicated three times within a two-month interval after the production of the ETs, and the results were expressed as mean ± standard deviation (s.d.).

### 2.5. Assessment of Entrapment Capacity

To quantify the entrapment of the active agents within the ET nanovesicles, the concentrations of CUR and PIP were assessed on the subsequent day post-production via ultrafiltration (Microcon centrifugal filter unit with YM-10 membrane, NMWCO 10 kDa, Sigma-Aldrich, St. Louis, MO, USA), followed by a high-performance liquid chromatography (HPLC) analysis. Precisely, 500 µL samples of ET–CUR and ET–PIP underwent ultrafiltration (Spectrafuge™ 24D Digital Microcentrifuge, Infitek Inc., Spokane, WA, USA) at a centrifugal force of 4000 rpm for a duration of 20 min. The retentate samples were then diluted in ethanol (1:10 *v*/*v*) and agitated with a magnetic stirrer for half an hour. An aliquot (100 µL) of the vesicular dispersion was also treated similarly to ascertain the actual drug content within the formulation. Prior to HPLC, the samples were passed through nylon syringe filters (0.22 µm). The entrapment capacity (EC) was calculated as:

EC = *D*/*T_D_* × 100 (1)

Here, *D* represents the amount of drug retained by the vesicles, and *T_D_* denotes the real drug content in the entire formulation.

### 2.6. Franz Cell Methodology

Franz diffusion cells with an orifice diameter of 0.9 cm (PermeGear Inc., Hellertown, PA, USA) were utilized for both the in vitro release test (IVRT) and the in vitro permeation test (IVPT). The IVRT employed PTFE membranes, while STRAT-M^®^ membranes were chosen for the IVPT. The synthetic PTFE membrane function was only to divide the upper and lower cell compartments as an inert support [40,41]. The STRAT-M^®^ membrane had, instead, the function of mimicking the *stratum corneum*, being made of two polyether sulfone layers overlapped by one polyolefin bottom layer impregnated with synthetic lipids [42]. Prior to their placement in the Franz cells, the membranes were hydrated in an ethanol:water mixture (50:50, *v*/*v*) for one hour. The receptor chamber was filled with 5 mL of the same ethanol:water solution to maintain sink conditions, stirred magnetically at 500 rpm, and temperature-regulated at 32 ± 1 °C. A volume of 1 mL of ET–CUR or ET–PIP, or their respective ethanolic solutions (ethanol:water 30:70, *v*/*v*, with a drug concentration of 0.25 mg/mL), was introduced into the donor compartment, which was then sealed to prevent solvent loss. During the test interval of 0–24 h, 500 µL samples from the receptor phase were periodically collected for HPLC drug quantification. Each sampled volume was replaced with a fresh medium. The drug concentrations were analyzed in six independent assays, and the mean values ± s.d. were determined.

### 2.7. In Vitro Release Tests (IVRTs)

For IVRT data interpretation, the released drug quantity (CUR or PIP in µg/cm^2^) was graphed against the square root of the elapsed time [43]. To discern the release kinetics of the drug from various formulations, we calculated ‘R’, the rate of cumulative drug release as a function of the square root of time, and ‘A’, the total amount of drug released at the final sampling point (8 h) [43]. Additionally, the released drug quantity was expressed as a percentage of the amount of drug loaded in the formulations. The mechanism of drug release was examined by applying a regression analysis to the cumulative percentage of drug release over time, according to models such as the zero-order kinetics, first-order kinetics (log cumulative % drug remaining vs. time), Higuchi and Peppas (log cumulative % drug released vs. log time) models. The suitability of the model fits was verified using the DDsolver add-in for Excel 2016 (Version 2312 Build 16.0.17126.20126).

### 2.8. In Vitro Permeation Tests (IVPTs)

For the IVPTs, the permeated amount of CUR and PIP (µg/cm^2^) was plotted over time [43,44]. Fick’s law was employed as the guiding principle, postulating steady-state drug permeation through the membrane under sink conditions, where the drug concentration in the receptor phase is negligible compared to the donor compartment. The steady-state flux, ‘Jss’, is defined as the rate of drug transport per unit area and is given by:

Jss = P × Cd × D/e(2)

Here, ‘P’ is the partition coefficient, ‘Cd’ represents the drug concentration in the donor compartment, ‘D’ is the diffusion coefficient for CUR/PIP and ‘e’ is the membrane thickness, as specified by the manufacturer [45]. From these values, the permeability coefficients, ‘Kp’, and lag times, ‘Tlag’, were derived using the steady-state portion of the drug’s cumulative penetration profile over time, as reported in the Supplementary Data section. In addition, the permeated amount of drug was expressed as a percentage of the amount of drug loaded in the formulations.

### 2.9. HPLC Analysis

The HPLC analyses were performed using Perkin Elmer Series 200 HPLC Systems (PerkinElmer, Waltham, MA, USA), equipped with a micropump, an auto sampler, and an UV detector operating at 360 nm and 243 nm for CUR and PIP, respectively. A stainless-steel C-18 reverse-phase column (15 × 0.46 cm) packed with 5 µm particles (Hypersil BDSC18 Thermo Fisher Scientific S.p.A., Milan, Italy) was eluted at a flow rate of 1 mL/min with a mobile phase containing methanol/water 80:20 *v*/*v*. The injection volume was 5 µL, and the retention times were 2.3 min for CUR and 2.7 min for PIP.

### 2.10. Ferric Reducing Antioxidant Power (FRAP Test)

The in vitro antioxidant activity of the ETs, drug-loaded ETs and drug solutions was investigated using the FRAP test, a colorimetric method for assessing the ability to reduce ferric ions (Fe^3+^) to ferrous ions (Fe^2+^) in an acidic environment, complexed with TPTZ (2,4,6-triridyl-s-triazine), as described by Benzie et al. [44]. In the presence of an antioxidant, the Fe(III)–TPTZ complex is reduced to its ferrous form (Fe(II)), which has an intense blue color with a maximum absorption peak at 593 nm. One point nine milliliters of FRAP reagent (a mixture of 0.1 M acetate buffer pH 3.6, 10 mmol/L TPTZ in 40 mmol/HCl and 20 mmol/L ferric chloride in a 10/1/1 ratio) was added to 0.1 mL of an appropriately diluted sample (or blank solvent). All samples were then incubated in the dark at 37 °C for 10 min, followed by a measurement of their absorbance at 593 nm with a UV-Vis spectrophotometer (UV-31 SCAN ONDA Spectrophotometer, Giorgio Bormac spectrophotometer Srl, Carpi (MO), Italy). Trolox is used as the standard for the calibration curve, and the results are expressed as µmol Trolox equivalent (TE) per gram of sample.

### 2.11. Patch Test

A study on human volunteers was conducted to assess the skin irritation potential of ET nanovesicles encapsulating CUR and PIP upon a single application to the skin. This investigation adhered to the standardized protocols for evaluating the skin compatibility of cosmetic ingredients that might be irritants, as outlined by SCCNFP/0245/99 [43]. The University of Ferrara’s Cosmetology Center, under an approved protocol by its Ethics Committee (study number: 170583), conducted this test on 20 healthy male and female volunteers, randomly selected, who consented in writing to participate, ensuring the exclusion of individuals with dermatological conditions, a history of allergic skin reactions or those currently undergoing anti-inflammatory treatments.

Each volunteer was administered 10 mg of the ET formulation, containing CUR and PIP, using aluminum Finn chambers (Bracco, Milan, Italy) affixed to the forearm or back with adhesive tape. Specifically, an insulin syringe was employed to deposit the samples directly into the chambers, maintaining contact with the skin for a period of 48 h. After the removal of the chambers and the cleansing of any residual substance, skin reactions, including erythema and/or edema, were assessed after 15 min and again after 24 h. Erythema was categorized into three levels: mild, readily apparent and moderate to severe. An average irritation index was computed by aggregating the scores of erythema and edema. This index was then interpreted on a scale that designates a score above 0.5 as indicative of slight irritation, scores between 2.5 and 5 as moderate irritation, and scores from 5 to 8 as representing significant irritation.

### 2.12. Biological Activity Studies

#### 2.12.1. Human Specimens

Human Caucasian skin explants were obtained from elective abdominoplasties from 3 different donors after the approval of the Institutional Biosafety (IBC) Committee at NC State University. Skin biopsies (12 mm) were collected with a punch, and the subcutaneous fat was removed with sterile scissors. The skin biopsies were washed in Phosphate Buffer Solution (PBS) and then transferred into 6-well plates prefilled with 1 mL of DMEM High Glucose supplemented with 10% Fetal Bovine Serum (FBS), 100 IU/mL penicillin and 100 mg/mL streptomycin (complete medium) using a sterile technique [45]. The samples were incubated at 37 °C in a 5% CO_2_/95% air atmosphere for overnight recovery.

#### 2.12.2. Treatment with Formulations and DEEExposure

After overnight recovery, the skin biopsy medium was replaced with 1 mL of fresh complete medium, and the samples were treated with the indicated formulations. Briefly, 10 µL of each formulation (0.25 mg/mL) was applied topically on the skin explants and spread using a sterile glass rod. For the combined treatments (ET PIP + ET CUR and SOL PIP + SOL CUR), 5 µL of each formulation was applied to the skin and evenly mixed. Some samples were kept untreated as controls. The plates were incubated in a humidified 5% CO_2_/95% air atmosphere for 60 min before exposure to the diesel engine exhaust (DEE) insult. For all the donors, the experiment was performed in triplicate for each condition. One hour after the first treatment, the skin biopsies were exposed to DEE for 60 min, as previously described [45], by using a Kubota RTV-X900 diesel engine (KUBOTA Corporation, Gainesville (GA), USA) (3-cylinder, 4-cycle diesel with overhead valves, 1123 cc that has 24.8 HP at 3000 rpm). The untreated control samples (CTRLs) were left in the incubator.

After DEE exposure, the tissue samples were incubated in a humidified 5% CO_2_/95% air atmosphere until the following day. The next day, treatment and exposure were repeated in the same way. After two days of exposure, the skin biopsies were left in the incubator for 30 min before their collection.

#### 2.12.3. Tissue Collection and Immunohistochemical Analysis

The skin explants were fixed in 10% neutral buffered formalin for 48 h at 4 °C, dehydrated and embedded in paraffin. Then, 5 µm-thick sections were deparaffinized in xylene and then rehydrated through a series of decreasing ratios of alcohols to water. The immunohistochemical analysis was performed as previously described [45]. The tissues were incubated with primary antibodies for 4HNE (dil. 1:400) (AB5605, Millipore Corporation, Burlington, MA, USA), type I collagen (dil. 1:200) (AB138492, Abcam, Cambridge, UK) and Filaggrin (dil. 1:50) (sc-66192, Santa Cruz Biotechnology, Inc., Dallas, TX, USA) in 0.25% BSA in PBS overnight at 4 °C, and with fluorochrome-conjugated secondary antibodies (Alexa Fluor 568, A11004 or Alexa Fluor 488, A11055) diluted 1:1000 in 0.25% BSA in PBS at room temperature for 60 min. Nuclei were stained with DAPI (dil. 1:50,000) (D1306, Invitrogen, Waltham, MA, USA) in PBS. Coverslips were mounted onto glass slides using Fluoromount-G™ Mounting Medium (00-4958-02, ThermoFisher Scientific, Waltham, MA, USA). The tissues were examined using a Zeiss Z1 AxioObserver LSM10 confocal microscope at 40× magnification, and the images were quantified using ImageJ software 1.53a (Java 1.8.0_172, National Institutes of Health, Bethesda, MD, USA).

### 2.13. Statistical Analysis

GraphPad Prism 9 (Version 9.4.1 (458), GraphPad Software Inc., La Jolla, CA, USA) was used to perform the statistical analysis. For each of the variables tested, an analysis of variance (1-way or 2-way ANOVA), followed by Tukey’s post hoc test, was assessed. The data are expressed as the mean ± SD of triplicate determinations obtained in three independent experiments, and a *p* value < 0.05 was considered statistically significant. For all the experiments, the control values were set to 1.0, and the other values were expressed as a fold change.

## 3. Results

### 3.1. Preparation of Ethosomes

ET nanovesicles were designed as biocompatible nanocarriers for the dermal and transdermal delivery of drugs. Their ability to allow drug penetration into the deeper layers of the skin is due to the presence of ethanol, which acts synergistically with PC as a penetration enhancer.

The bulk approach we previously employed for ET preparation was a cold method based on the dropwise addition of water to a PC–ethanol solution (30 mg/mL) kept under magnetic stirring (Figure S1a). From previous studies, 0.9% *w*/*w* of PC and 30% *v*/*v* of ethanol was the composition selected, enabling us to obtain vesicles whose Z-average diameter was around 200 nm with a dispersity index below 1.2, suggesting a homogeneous size distribution.

In this study, we evaluated the microfluidic method as a possible alternative production protocol for ETs [46,47]. The device was based on two syringe pumps connected to a chip with two crossed microchannels, two flow regulators and an optical microscope for control (Figure S1b).

The LP and AP flows along the axis of the channel meet at the intersection of the two channels of the microfluidic chip. The diffusion of water and ethanol at the ethanol/water interface results in PC self-assembly, producing vesicles of precise and controlled sizes in a reproducible manner. The ETs appear as homogeneous milky dispersions, free from sedimentation phenomena.

Table 1 summarizes the microfluidic parameters employed for the ET preparation. The FRR was always 2:1 (corresponding to ethanol 33%, *v*/*v*), selected to maintain almost the same PC concentration and ethanol volume ratio selected in our previous ET investigations, while the TFR was between 12 and 90 μL/min.

The evaluation of the size distribution parameters by PCS evidenced that the TFR affected the Z average; particularly, the lowest TFR value led to the smallest mean diameter and D.I., while increasing the TFR values led to larger vesicles and a more heterogeneous size distribution (Table 1 and Figure S2). Considering the two ET production procedures, the bulk methodology was found to be more convenient than microfluidics. In fact, the first method enables us to obtain 5 mL of ETs with a diameter of approximately 200 nm in 30 min. Conversely, to obtain ETs with a comparable size by the microfluidic method, a smaller TFR should be employed, which in 30 min would produce a 360 μL ET volume, i.e., 14-fold smaller than the bulk method. For this reason, in place of a possible scaling-up protocol, we selected the bulk cold method for ET production.

### 3.2. Drug Loading in Ethosomes

ET–PIP and ET–CUR were obtained by the previously described solubilization of the drug in the PC–ethanol solution, resulting in homogeneous milky dispersions, showing a characteristic yellow color in the case of ET–CUR. Remarkably, the simple cold method enabled us to efficiently load either CUR or PIP in the ETs, while the combination of both the phytocompounds (0.25 mg/mL) in the same dispersion resulted in drug sedimentation.

Table 2 reports the composition and size distribution parameters of ET and drug-loaded ET produced by the bulk cold method.

The ET–PIP and ET–CUR Z-average mean diameters, as reported in Table 3, were superimposable and slightly larger than those of the ET vesicles. The dispersity index was always below 0.2, indicating homogeneous size distributions. After 2 months of storage, a slight Z-average increase was detected (Table 3).

**Figure 1:**
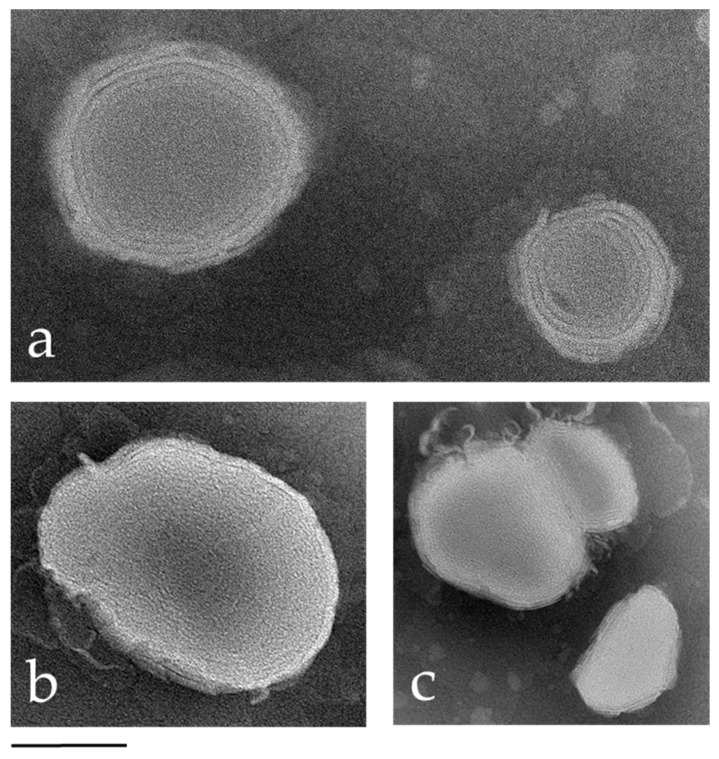
Transmission electron micrographs of ETs (**a**), ET–CUR (**b**) and ET–PIP (**c**). The bar corresponds to 150, 100 and 200 nm in panels (**a**), (**b**), (**c**), respectively.

In order to obtain information about the morphology of the nanosystems, the ETs and drug-loaded ETs were visualized by TEM. Figure 1 displays micrographs showing spherical or ovoid nanovesicles, remarkably exhibiting atypical inner multilamellar organization. The presence of CUR (Figure 1b) or PIP (Figure 1c) did not affect the nanovesicle morphology.

#### Drug Entrapment Capacity

The EC was evaluated through the ultrafiltration method, which allows us to separate the vesicular fraction from the aqueous dispersing phase. Subsequently, the vesicular fraction was diluted in ethanol to promote vesicle disaggregation. The HPLC analysis allowed us to quantify the associated drug.

As shown in Table 4, in both cases, most of the drug is associated with the nanovesicles. The EC was 1.22-fold higher in the case of ET–CUR with respect to ET–PIP. This diversity may be related to the different drug physicochemical parameters (Table S1). Indeed, CUR is characterized by a slightly higher molecular weight and lipophilicity with respect to PIP, suggesting that CUR could possibly be more efficiently retained within the PC double layers of the ET vesicles [48,49].

The simultaneous entrapment of CUR and PIP in the ETs was not achievable, possibly because at the employed concentration (0.25 mg/mL), the drugs in the PC–ethanolic solution exceeded the point of saturation. Nonetheless, the co-administration of both ET formulations could result in a possible improvement in the drug efficacy related to the application of the ET formulation embodying the individual drugs.

### 3.3. Evaluation of Antioxidant Activity

To evaluate the effect of CUR loading in ETs and its association with PIP loading in ETs, ET–CUR (CUR 0.25 mg/mL), ET–PIP (PIP 0.25 mg/mL) and their respective drug solutions were evaluated in terms of their ferric ion reduction potential (the FRAP test). In addition, empty ETs were assayed, as were the combinations of ET–PIP + ET–CUR (PIP: 0.125 mg/mL; CUR: 0.125 mg/mL) and SOL–PIP + SOL–CUR at the same drug concentration. The results reported in Table 5 show that the ETs, ET–PIP and SOL–PIP have no antioxidant activity, whereas the well-known antioxidant activity of CUR was detected (*p* > 0.6) [50] with very similar values in the vesicular and in-solution formulations.

From a practical point of view, since in the FRAP assay, SOL–CUR and ET–CUR were diluted in the same solvent, the antioxidant activity of CUR was expected to be the same for the two samples, demonstrating that the drug loaded in the ETs did not undergo any chemical degradation. The behavior of CUR as an antioxidant or pro-oxidant depends on its structural form; indeed, the molecule exists in two tautomeric forms: keto and enol. In the keto form, CUR exerts antioxidant activity, while in the enol form, it is subject to degradation [10]. The acidic environment of the FRAP assay favored the keto form equally for CUR in the solution or loaded in ETs. Therefore, CUR, either in solution or loaded in ETs, was directly available to participate in the reaction occurring in the assay, which involved the reduction of Ferric iron (Fe^3+^) to Ferrous iron (Fe^2+^).

The combination of the two active ingredients in the solution showed no change in the antioxidant power of CUR, whereas the combination of the vesicular ET–PIP and ET–CUR formulations resulted in a statistically significant (*p* < 0.035) increase in its antioxidant power compared to ET–CUR. The FRAP in vitro results allow us to state that the combination of ET–PIP and ET–CUR boosts the antioxidant effect of CUR, confirming the evidence published in numerous studies [50,51].

### 3.4. IVRT

An in vitro Franz cell system was employed to evaluate the ETs’ capability to control CUR and PIP release. The synthetic PTFE membrane was chosen to separate the upper and lower compartments, acting as an inert support that does not affect the drug release. The release kinetics of CUR and PIP, either loaded in ETs or in ethanol solutions, were compared.

**Figure 2:**
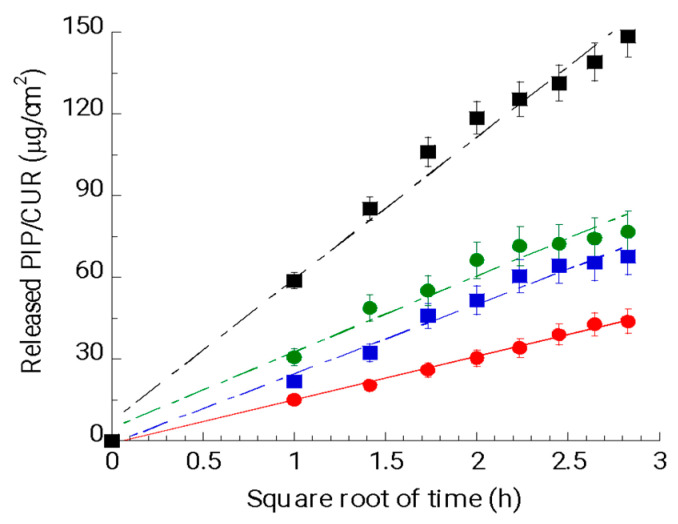
CUR and PIP release kinetics from ET–CUR (red circles), SOL–CUR (blue squares), ET–PIP (green circles) and SOL–PIP (black squares), as determined by Franz cells associated with the PTFE membrane. Data are the mean of 6 independent experiments ± s.d.

As expected, the ETs were able to control both CUR and PIP release better as compared to the drug solutions (Figure 2 and Figure S3). In general, CUR was released more slowly than PIP, both in the ET and solution formulations.

The percentage release profile of the drugs, expressed as a percentage of the drug released, as reported in Figure S3’s plot, confirms the trend obtained by plotting the mass units (μg) per unit area (cm^2^) and revealed that the percentage of drug release with respect to the drug loaded in the formulation was 57%, 33%, 24% and 15% in the cases of SOL–PIP, ET–PIP, SOL–CUR and ET–CUR, respectively.

A zero-order plot, first-order plot, Higuchi plot and Peppas plot were applied in order to elucidate the mechanism of drug release from the ETs [52]. As reported in Table 6, comparing R^2^ values, CUR and PIP release followed Higuchi kinetics due to the initially controlled drug diffusion through the matrix, as found by other authors [53]. The fitting into the Peppas equation revealed a Fickian diffusion mechanism with a release exponent (n) of around 0.5.

### 3.5. IVPT

Franz cells associated with the synthetic STRAT-M^®^ membrane were used to evaluate ETs’ effect on CUR and PIP permeation through the skin. The architecture of STRAT-M^®^, consisting of different synthetic layers impregnated with lipids (Section 2.6), confers on the membrane a peculiar structure able to mimic skin hydrophobicity and porosity. Indeed, STRAT-M^®^ can be considered a valuable model for *stratum corneum* barrier properties [42,54]. Drug permeation across the skin occurs in two main steps. The first one is described by the P coefficient, which reflects the preferred distribution of the drug in the skin/membrane or vehicle. The second step, described by the D and Kp coefficients, refers to the diffusion of the drug through the skin (or membrane), depending on its characteristics and vehicle properties [43,55,56].

**Figure 3:**
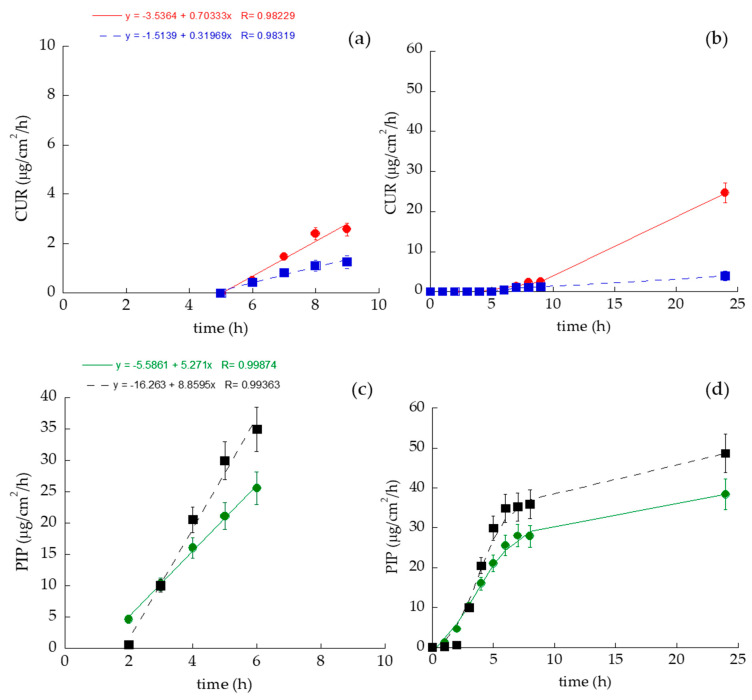
CUR (**a**,**b**) and PIP (**c**,**d**) permeability kinetics from ET–CUR (red circles), SOL–CUR (blue squares), ET–PIP (green circles) and SOL–PIP (black squares), as determined by Franz cells associated with the Strat-M membrane. (**a**,**c**) Panels show the linear part of the kinetic profiles; (**b**,**d**) Panels show diffusion profile over 0–24 h. Data are the mean of 6 independent experiments ± s.d.

Figure 3 and Figure S4 show the CUR (a,b) and PIP (c,d) diffusion profiles through the STRAT-M^®^ membrane, while Table 7 reports the diffusion parameters.

In general, the diffusion kinetic profiles were much faster in the case of PIP with respect to CUR. A lag time was detected in all the diffusion kinetics, which was longer for CUR with respect to PIP, particularly in the case of ET–CUR (five-fold higher than ET–PIP), confirming a stronger CUR association with the vesicular system. In the case of CUR, the drug diffused faster from the ETs with respect to the solution, with an increase in drug diffusion after 9 h. Conversely, in the case of PIP, the drug diffusion was faster in the solution SOL–PIP with respect to ET–PIP, with a slope decline after 9 h. The Kp values followed the order SOL–PIP > ET–PIP > ET–CUR > SOL–CUR. Particularly in the case of ET–PIP, the Kp value was 7.4-fold higher with respect to ET–CUR. The highest D value was found in the case of ET–PIP, around five-fold higher with respect to ET–CUR. Remarkably, the *p* values suggest in the case of ET–CUR a higher drug partition towards the membrane with respect to the solution, while an opposite behavior was found in the case of PIP, suggesting that CUR loading in ETs could promote a drug interaction towards the membrane mimicking the skin.

### 3.6. In Vivo Comparative Irritation Test

In order to evaluate in vivo if ET–CUR and ET–PIP could induce an irritative reaction when applied to the skin, a patch test was performed on 20 health volunteers. The number of irritative reactions occurring 15 min and 24 h after the removal of the Finn Chamber was recorded and expressed as irritation indexes. It is noteworthy that the average irritation index score induced by the single application of ET–CUR or ET–PIP was 0.1, while the score obtained by the application of both formulations was 0.2, as expected. Since in all cases the reaction was negligible, the formulations can be classified as not irritating for the human skin, either if applied singularly or in combination.

### 3.7. Biological Activity Studies

#### Immunohistochemical Analysis

**Figure 4:**
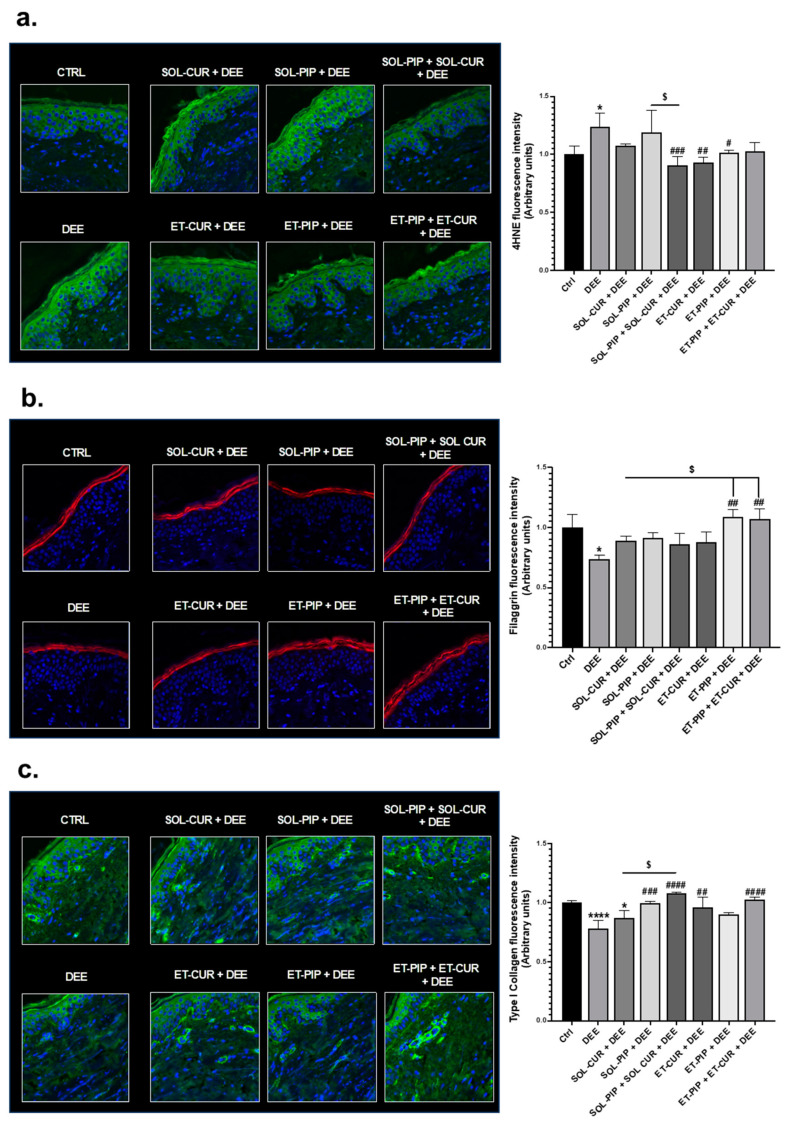
Immunofluorescence staining for 4HNE (**a**) filaggrin (**b**) and type I collagen (**c**) in human skin biopsies treated with the indicated formulations and exposed to DEE. Red or green staining represents the selected proteins, and the blue staining (DAPI) represents nuclei. Images were taken at 40× magnification. The fluorescent signal of the different markers was quantified using ImageJ software 1.53a (Java 1.8.0_172) and is expressed in the graphs. Data are the results of the averages of at least three different experiments, with *^,#,$^ *p* < 0.05; ^##,^ *p* < 0.005; ^###,^ *p* < 0.001; and ^****,####,^ *p* < 0.0001, by 2-way ANOVA followed by Tukey’s post hoc comparison test [57]. DEE, SOL–CUR + DEE, SOL–PIP + DEE, SOL–PIP + SOL–CUR + DEE, ET–CUR, ET–PIP, ET–PIP + ET–CUR + DEE vs. Ctrl (*); CTRL, SOL–CUR + DEE, SOL–PIP + DEE, SOL–PIP + SOL–CUR + DEE, ET–CUR, ET–PIP, ET–PIP + ET–CUR + DEE vs. DEE (^#^); SOL–PIP + SOL–CUR + DEE vs. SOL–CUR + DEE (^$^).

Human skin biopsies were employed to evaluate the protective effect of the selected formulations in terms of the cutaneous oxidative and structural damage induced by DEE. For this purpose, the skin explants were pre-treated with the different formulations and exposed to DEE. As shown in Figure 4a, the exposure to DEE induced a significant increase in 4-hydroxy-nonenal (4HNE) levels, a reactive aldehyde originating from lipid peroxidative events, in the human skin explants compared to the controls. Almost all the formulations prevented oxidative damage by reducing the levels of 4HNE at basal conditions, and in particular, ET–CUR, ET–PIP and the combination of the drug solutions SOL–PIP + SOL–CUR were found to be the most effective formulations. The cutaneous structural damage was evaluated by measuring the expression levels of filaggrin, a protein involved in the skin differentiation process and an important member of the skin cornified cell envelope. Although the skin explants exposed to DEE displayed reduced levels of filaggrin, indicating structural damage promoted by the pollutant, the treatment with the different formulations, especially ET–PIP and the combination of ET–PIP + ET–CUR, efficiently counteracted the DEE-induced skin damage (Figure 4b). In addition, besides SOL–CUR, all the formulations could prevent the degradation of type I collagen (Figure 4c), an essential protein that confers elasticity and strength to the skin and is degraded during aging.

The combination of CUR and PIP, both as SOL–PIP + SOL–CUR and as ET–CUR + ET–PIP, was the most efficient in preventing the damage, suggesting that PIP might act as a biopotentiator of CUR to counteract the skin aging process induced by air pollutants.

## 4. Discussion

ETs have been engineered as biocompatible carriers for the dermal and transdermal administration of drugs. With respect to other nanoparticulate systems, such as liposomes, ETs can effectively load large amounts of lipophilic drugs while maintaining their stability. In addition, the inclusion of ethanol aids in enhancing drug penetration into the skin’s deeper layers, working in synergy with PC to boost drug permeability [58,59,60]. Initially, we explored the fabrication of ETs using microfluidics—a technique traditionally applied to nanoparticle, nanoemulsion and liposome preparation—due to its precise control over particle size and drug encapsulation efficiency. However, the established bulk method [35,36,54,61,62,63], which involves hydrating a PC–ethanol mixture with water under magnetic stirring, proved to be more efficient concerning time and volume output, at least with the specific FRR and cross-junction microchip utilized in our experiments.

A significant advantage of ETs is their ability to form nanovesicles with a stable mean diameter of 150–200 nm, which remains consistent for at least two months (Table 1 and Figure S2). This is in stark contrast to liposomes, which often have a heterogeneous size distribution, necessitating an extrusion method for their size reduction. Through the bulk cold method, both CUR and PIP are effectively encapsulated within ETs (Table 4), with CUR exhibiting a slower release and higher retention within the vesicles compared to PIP (Figure 2 and Figure S3, Table 6).

The IVPTs indicated that CUR-loaded ETs enhance drug permeability more than their solution counterpart. However, for PIP, its loading into ETs did not significantly impact its permeability, despite PIP demonstrating a faster permeation rate than CUR, as evidenced by its higher diffusion coefficient in the ET–PIP systems (Figure 3 and Figure S4, Table 7).

The FRAP assay results suggest that ET–PIP and ET–CUR could be combined to mitigate skin conditions associated with oxidative stress (Table 5). This in vitro test does not involve biological specimens or a physiological environment. When tested in a biological environment, we need to consider the bioavailability of the drug. For instance, the ex vivo analysis conducted on the human skin explants showed that SOL–CUR cannot properly pass the *stratum corneum*, so itis not available to exert its antioxidant properties. On the contrary, when delivered via ETs, CUR can reach the skin layers and be available for the cells, explicating its antioxidant activity. Moreover, the FRAP test pointed out that, in vitro, the combination of ET–CUR and ET–PIP enhances the antioxidant effect of CUR. The greater permeability of loaded CUR and its slower release suggest that the skin administration of ET–CUR could promote a prolonged protective effect, while the combination of ET–PIP could potentiate CUR action against secondary responses to oxidative stress in the skin.

Notably, ex vivo studies have shown that human skin exposed to DEE exhibits a marked increase in 4HNE levels, indicating oxidative stress (Figure 4a).

Indeed, exposure to environmental pollutants is known to cause oxidative and inflammatory reactions within the human skin that can ultimately result in skin structural damage and premature aging [64]. ROS induced by air pollutants can promote lipid peroxidative events that culminate in the production of secondary mediators, such as the reactive aldehyde 4HNE, that can propagate cutaneous oxidative damage throughout the skin layers [65]. 4HNE can affect skin proteins’ functionality by forming adducts that cause such proteins to lose their activity and be degraded [65]. As a matter of fact, the ex vivo studies demonstrated that the human skin exposed to both SOL–PIP and SOL–CUR failed to prevent oxidative damage from DEE, aligning with previous findings of SOL–PIP’s lack of antioxidant activity and SOL–CUR’s poor permeability. Conversely, both the ET–CUR and ET–PIP formulations reduced the 4HNE levels to baseline, with ET–CUR showing a more pronounced protective effect (Figure 4a). These results are in line with previous work, where ETs have been found to be able to reach the deeper layers of the skin, thus allowing the release of a drug within the cutaneous tissues [36]. The TEM analysis revealed the presence of these vesicular nanosystems in the connective tissue of the upper papillary dermis that underlies the epidermis of the human skin explants treated for 3 h with ETs.

Of note, CUR has been shown to protect from lipid peroxidative events, behaving as a chain-breaker antioxidant [66]. Indeed, the functional groups present in CUR’s structure, such as the β-diketo group, carbon and phenyl rings, and carbon–carbon double bonds, are able to donate H-atoms, thus protecting biomembranes against peroxidative damage [67,68]. Although PIP was also demonstrated to possess antioxidant properties by quenching ROS, free radicals and reactive metabolic intermediates, its antioxidant activity is strictly related to the dose used [19,20,69]. Thus, we believe that at this concentration, PIP was not able to exert an evident antioxidant property, whereas CUR prevented the lipid peroxidation cascade occurring within the skin upon DEE exposure.

Moreover, considering the slower CUR diffusion in ET–CUR, it is plausible that its sustained release confers a prolonged protective effect against secondary oxidative stress responses in the skin [64]. Oxidative stress is typically an immediate response to an insult, but the ensuing cascade can lead to a sustained skin response. Moreover, DEE exposure is associated with reduced levels of filaggrin, a key protein in the skin’s outer layer essential for maintaining structural integrity, hydration and cutaneous barrier properties.

The oxidative and inflammatory events promoted by air pollutants can alter the skin cell differentiation and proliferation processes, affecting skin structural proteins and thus skin integrity and functionality, and rendering the cutaneous tissue more susceptible to being affected by other environmental insults [64,70,71]. The treatment of the skin with natural antioxidant and anti-inflammatory compounds was found to counteract the air pollutants’ damage and restore skin homeostasis [45,70,72,73]. In this context, natural antioxidants and anti-inflammatory agents have been shown to counteract pollutants’ effects and restore skin equilibrium [74,75].

In our study, the ET formulations effectively prevented filaggrin degradation due to DEE (Figure 4b). The combination of ET–PIP and ET–CUR was particularly effective, suggesting that while CUR alone did not demonstrate protective effects, the presence of PIP may have enhanced CUR’s efficacy at the selected dose. CUR, indeed, is known to exert anti-inflammatory properties by suppressing prostaglandin (PG) synthesis, interfering with the signaling mechanisms involved in Cyclooxigenase-2 (COX-2) enzyme transcription and inhibiting the Lipoxygenase (LOX) pathway [66]. Moreover, CUR has been shown to modulate the activation of the nuclear factor kappa-light-chain-enhancer of activated B cells (NF-κB) by preventing its translocation to the nucleus, which is necessary for the transcription of genes involved in the inflammatory response. Indeed, NF-κB is usually sequestered in the cytoplasm by a family of inhibitors called IκBs (Inhibitor of κB), whose phosphorylation by the IκB kinase (IKK) promotes their degradation and the release of NF-κB. Several studies demonstrated that CUR could inhibit the activation of IκB kinase (IKK), thus preventing IκB phosphorylation and blocking the translocation of NF-κB within the nucleus [66,76,77,78]. Considering the strict link between oxidative and inflammatory responses, CUR has been shown to regulate oxidative stress via both ROS and NF-κB pathways [79].

PIP, as well, has been reported to be a potent inhibitor of NF-κB translocation in melanoma [80] and to attenuate cigarette-smoke-induced oxidative stress and lung inflammation by upregulating Sirtuin1 (SIRT1) and promoting the inhibition of NF-κB and the activation of the antioxidant response via nuclear factor erythroid 2-related factor 2 (Nrf2) [81].

It should be mentioned that skin structural damage is often a consequence of a combination of oxidative and inflammatory reactions; therefore, it is possible that CUR at the dose applied in this study is not able to exert a protective role, whereas PIP might contribute to the anti-inflammatory response of the skin. Moreover, PIP’s permeability and diffusion were higher in the case of ET–PIP with respect to ET–CUR, suggesting its capability to counteract structural damage earlier than CUR under our experimental conditions. It is worth recalling that, whereas ROS production is a quick event occurring right after exposure to environmental insults, cutaneous structural damage is the result of a later response of the skin to ox-inflammatory damage. The slower drug diffusion and permeability in the case of ET–CUR could account for its protective effect displayed after a prolonged exposure time. Further experiments are needed to better elucidate the possible protective effect of ET–CUR on chronic exposure.

Finally, we observed that all the formulations combated type I collagen loss induced by DEE, a key component of the skin’s elasticity and strength (Figure 4c). The combined treatments of CUR and PIP appeared to have a synergistic effect in preserving collagen, suggesting their potential in protecting against the skin inflammation process [82].

Indeed, the premature skin aging process induced by exposure to air pollutants is mainly accompanied by a loss of proteins that confer elasticity and mechanical strength to the skin, such as type I collagen. Indeed, the activation of Metalloproteinases (MMPs) in the extracellular matrix in response to environmental stressors can promote the degradation of this protein, promoting wrinkle formation and other signs of aging [83].

Notably, CUR can promote type I collagen induction via the inhibition of ROS [84] and modulate MMP activation, as can PIP [80,85].

Taken together, our results show that the combination of CUR and PIP may be helpful in regulating many inflammatory and oxidative stress pathways, thus broadly protecting the skin from detrimental external stimuli.

## 5. Conclusions

Considering that skin aging is a result of prolonged exposure to air pollutants, the use of drug-loaded ETs might represent the best approach to promoting a prolonged transdermal release of the drugs within the skin with respect to plain drug solutions, resulting in better protection against skin aging in the long term. Anyway, further ex vivo studies will be required to confirm this evidence, possibly evaluating the effect of ET–CUR and ET–PIP, either singularly or in combination, in protecting the skin against other environmental pollution stressors, such as ozone and UV exposure. Moreover, the possibility of administering, on a single nanoplatform, efficacious amounts of both CUR and PIP will be evaluated.

## Figures and Tables

**Table 1:** Ethosomes’ microfluidic conditions and size distribution parameters, as measured by PCS.

TFR ^a^ (μL/min)	FRR ^b^	F_AP_^c^ (μL/min)	F_LP_^d^ (μL/min)	PC ^e^ (%, *w*/*w*)	Ethanol (%, *w*/*w*)	Water (%, *w*/*w*)	Z Average ^f^ ± s.d. (nm)	D.I. ^g^ ± s.d.
12	2:1	4 + 4	4	0.9	29.1	70	192.30 ± 25.33	0.129 ± 0.013
24	2:1	6 + 6	6	0.9	29.1	70	236.40 ± 47.65	0.203 ± 0.032
36	2:1	12 + 12	12	0.9	29.1	70	296.17 ± 20.76	0.379 ± 0.013
60	2:1	20 + 20	20	0.9	29.1	70	308.01 ± 60.85	0.285 ± 0.119
90	2:1	30 + 30	30	0.9	29.1	70	282.61 ± 27.42	0.231 ± 0.048

^a^: total flow rate; ^b^: flow rate ratio (F_AP_/F_LP_), i.e., volumetric aqueous phase/lipid phase ratio; ^c^: aqueous phase flow; ^d^: lipid phase flow; ^e^: soy phosphatidylcholine; ^f^: mean diameter; ^g^: dispersity index; s.d.: standard deviation.

**Table 2:** Composition of ETs produced by the bulk method.

Formulation	PC ^1^ (%, *w*/*w*)	Ethanol (%, *w*/*w*)	Water (%, *w*/*w*)	PIP ^2^ (%, *w*/*w*)	CUR ^3^ (%, *w*/*w*)
ET	0.9	29.100	70	-	-
ET–PIP	0.9	29.075	70	0.025	-
ET–CUR	0.9	29.075	70	-	0.025

^1^: soy phosphatidylcholine; ^2^: piperine; ^3^: curcumin; standard deviation.

**Table 3:** Size distribution parameters of ethosomes as determined by PCS.

Formulation	Time (Days)	Z-Average (nm) ± s.d.	Dispersity Index ± s.d.
ET	1	206.32 ± 33.22	0.144 ± 0.012
	60	230.36 ± 23.11	0.155 ± 0.032
ET–PIP	1	213.13 ± 13.18	0.129 ± 0.019
	60	269.8 ± 15.13	0.196 ± 0.069
ET–CUR	1	213.03 ± 8.25	0.127 ± 0.046
	60	242.85 ± 9.97	0.189 ± 0.00

s.d.: standard deviation; data are the mean of 3 independent determinations on different batches.

**Table 4:** Entrapment capacity of the indicated formulations.

Formulation	EC ^1^ %
ET–PIP	78.95 ± 6.47
ET–CUR	96.87 ± 4.31

^1^: Entrapment capacity, as defined in Equation (1); data are the mean of 6 independent experiments ± s.d.

**Table 5:** Antioxidant activity of the indicated formulations.

Formulation	FRAP ^1^ (µmol TE/g)
ET	n.a. ^1^
SOL–PIP	n.a. ^1^
ET–PIP	n.a. ^1^
SOL–CUR	3042.86 ± 191.73
ET–CUR	3097.27 ± 112.83 *
SOL–PIP + SOL–CUR	2996.50 ± 95.54
ET–PIP + ET–CUR	3408.87 ± 68.80 *

^1^: not active; each value is the mean of at least three different experiments (mean ± SEM). * *p* < 0.035.

**Table 6:** IVRT parameters and kinetic data of the indicated formulations.

Formulation	R ^1^ (µg/cm^2^/h)	A ^2^ (µg/cm^2^)	Zero-Order Plot (R^2^)	First-Order Plot (R^2^)	Higuchi Plot (R^2^)	Peppas Plot (n/R^2^)
ET–CUR	15.961 ± 2.52	43.87 ± 8.28	0.935	0.947	0.996	0.54/0.995
SOL–CUR	25.67 ± 5.41	67.75 ± 5.50	-	-	-	-
ET–PIP	27.88 ± 6.04	76.65 ± 7.52	0.805	0.843	0.967	0.43/0.954
SOL–PIP	52.08 ± 4.24	148.49 ± 10.45	-	-	-	-

^1^: release rate; ^2^: amount of CUR/PIP released after 8 h; CUR and PIP concentrations were 0.25 mg/mL; data are the mean of 6 independent Franz cell experiments ± s.d.

**Table 7:** IVPT parameters of the indicated formulations.

IVPT Parameters	ET–CUR	SOL–CUR	ET–PIP	SOL–PIP
Jss ^1^ (μg/cm^2^/h)	0.70 ± 0.21	0.32 ± 0.1	5.27 ± 2.2	8.85 ± 3.1
Kp ^2^ (cm/h) × 10^3^	3.12 ± 0.94	1.28 ± 0.40	23.11 ± 9.65	43.81 ± 15.35
Tlag ^3^ (h)	5.03 ± 0.81	4.74 ± 0.33	1.06 ± 0.052	1.84 ± 0.23
D ^4^ (cm^2^ h^−1^) × 10^5^	3.33 ± 0.54	3.53 ± 0.25	15.80 ± 0.76	9.10 ± 1.14
P ^5^ membrane/vehicle	2.97 ± 1.37	1.15 ± 0.44	4.64 ± 2.16	15.26 ± 7.25
A ^6^ (µg/cm^−2^)	24.67 ± 4.1	3.92 ± 0.42	38.40 ± 5.67	48.66 ± 7.44

^1^: steady-state flux per unit area; ^2^: permeability coefficient; ^3^: lag time; ^4^ diffusion coefficient; ^5^ partition coefficient; ^6^ cumulative amount of DMF diffused at 24 h; CUR and PIP concentrations were always 0.25 mg/mL; data are the mean of 6 independent Franz cell experiments ± s.d.

## Data Availability

The data presented in this study are available on request from the corresponding author. The data are not publicly available due to privacy restrictions.

## Supplementary Materials

The following supporting information can be downloaded at https://www.mdpi.com/article/10.3390/antiox13010091/s1, S1: In vitro permeation test (IVPT) parameters; Table S1. Physicochemical parameters of curcumin (CUR) and piperine (PIP). Figure S1. Devices employed for ET production by bulk cold method (a) or microfluidics (b). The insets, respectively, show the dropping of water phase into the PC solution (panel (a)) and the microfluidic microchip (panel (b)); Figure S2. Effect of TFR on Z-average (─□─) and D.I. (- -∆ - -), as measured by PCS. Data are the mean of 6 independent experiments ± s.d.; Figure S3. CUR and PIP release kinetics from ET–CUR (red circles), SOL–CUR (blue squares), ET–PIP (green circles) and SOL–PIP (black squares), as determined by Franz cells associated to PTFE membrane. The percentage refers to the amount of drug loaded in the formulations. Data are the mean of 6 independent experiments ± s.d.; Figure S4. CUR (a) and PIP (b) diffusion kinetics from ET–CUR (red circles), SOL–CUR (blue squares), ET–PIP (green circles) and SOL–PIP (black squares), as determined by Franz cells associated with the Strat-M membrane. Data are the mean of 6 independent experiments ± s.d. The percentage refers to the amount of drug loaded in the formulations. Data are the mean of 6 independent experiments ± s.d.
