# Supplementary material for: Ethosomes for Curcumin and Piperine Cutaneous Delivery to Prevent Environmental-Stressor-Induced Skin Damage
Source: Antioxidants (Basel). 2024 Jan 11;13(1):91. doi: 10.3390/antiox13010091 (PMC10812558; doi:10.3390/antiox13010091)
Supplement: Supplementary file 1 [file antioxidants-13-00091-s001.zip › antioxidants-2808043-supplementary.pdf]

Supplementary data

Ethosomes for Curcumin and Piperine Cutaneous Delivery to Prevent Environmental-Stressor-Induced Skin Damage

Francesca Ferrara [1], Agnese Bondi [1], Walter Pula [1], Catia Contado [1], Anna Baldisserotto [2], Stefano Manfredini [2], Paola Boldrini [3], Maddalena Sguizzato [1], Leda Montesi [4], Mascia Benedusi [5], Giuseppe Valacchi [5,6,7,]* and Elisabetta Esposito [1,]*

[1] Department of Chemical, Pharmaceutical and Agricultural Sciences, University of Ferrara, 44121 Ferrara, Italy; frrfnc3@unife.it (F.F.); agnese.bondi@unife.it (A.B.); walter.pula@unife.it (W.P.); catia.contado@unife.it (C.C.); sgzmdl@unife.it (M.S.)
[2] Department of Life Sciences and Biotechnology, University of Ferrara, 44121 Ferrara, Italy; bldnna@unife.it (A.B.); stefano.manfredini@unife.it (S.M.)
[3] Center of Electron Microscopy, University of Ferrara, 44121 Ferrara, Italy; paola.boldrinicme@unife.it
[4] Cosmetology Center, University of Ferrara, 44121 Ferrara, Italy; leda.montesi@unife.it
[5] Department of Neurosciences and Rehabilitation, University of Ferrara, 44121 Ferrara, Italy; mascia.benedusi@unife.it
[6] Animal Science Department, NC Research Campus, Plants for Human Health Institute, NC State University, Kannapolis, NC 28081, USA
[7] Department of Food and Nutrition, Kyung Hee University, Seoul 26723, Republic of Korea
* Correspondence: giuseppe.valacchi@unife.it (G.V.); ese@unife.it (E.E.)

S1. In Vitro Permeation Test (IVPT) Parameters

The steady-state flux 'Jss' is defined as the rate of drug transport per unit area and is given by:

$$Jss = P \times Cd \times D/e$$

Here, 'P' is the partition coefficient, 'Cd' represents the drug concentration in the donor compartment, 'D' is the diffusion coefficient for CUR/PIP, and 'e' is the membrane thickness as specified by the manufacturer [45]. From these values, the permeability coefficients 'Kp' and lag times 'Tlag' were derived, using the steady-state portion of the drug's cumulative penetration profile over time.

$$Kp = Jss/Cd$$

The D value was calculated from T_{lag} according to Equation (S1):

$$T_{lag} = e^2/6 \text{ x } D \quad (S1)$$

Finally, P was calculated considering the Equation (S2):

$$Kp = D \text{ x } P/e \quad (S2)$$

Table S1. Physical-chemical parameters of curcumin (CUR) and piperine (PIP)

	**Drug**	
	CUR	PIP
**Molecular weight (g/mol)**	368.4	285.34
**LogP**	≈ 3	2.78

a

b

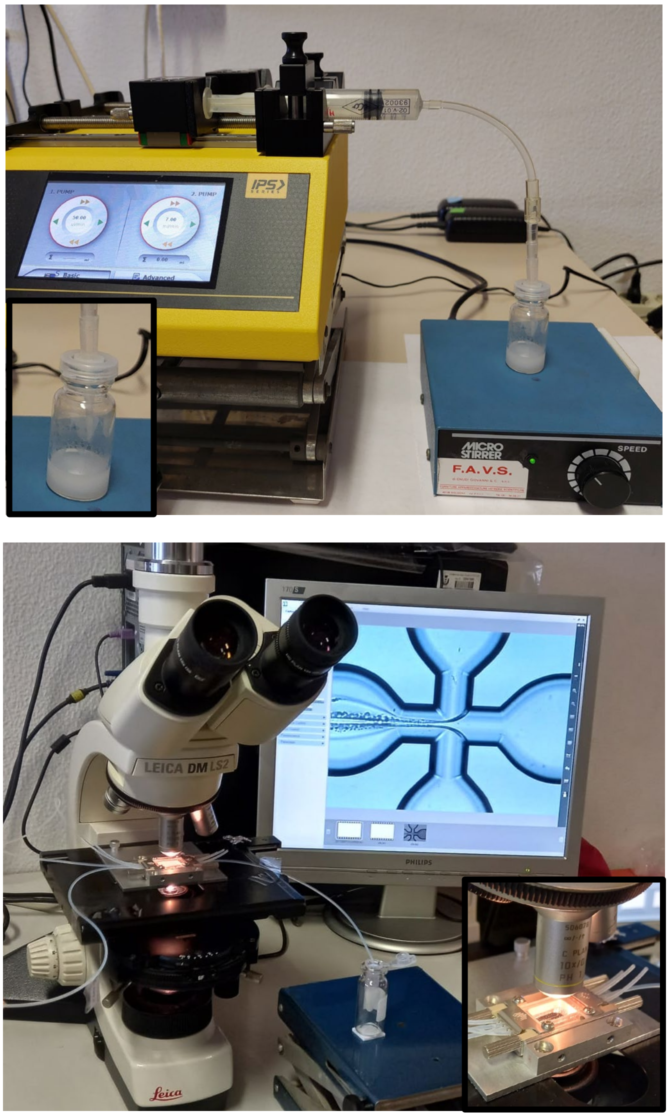

Figure S1. Devices employed for ET production by bulk cold method (a) or microfluidics (b). The insets respectively shows the dropping of water phase into the PC solution (panel (a)), and the microfluidic microchip (panel (b)).

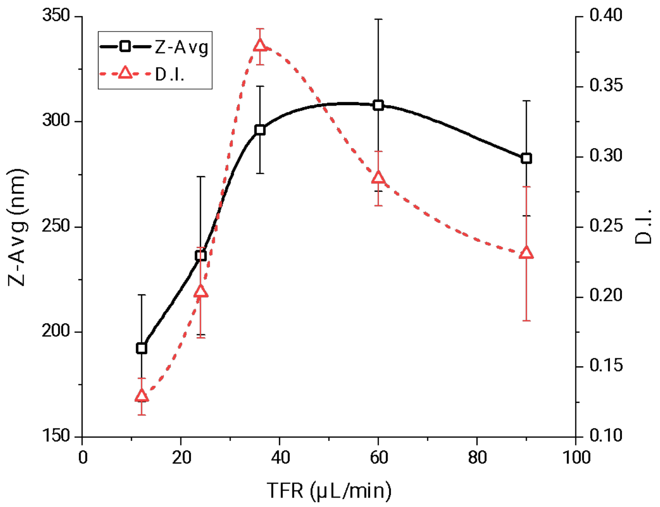

Figure S2. Effect of TFR on Z-Average (—□—) and D.I. (- -Δ- -), as measured by PCS. Data are the mean of 6 independent experiments ± s.d.

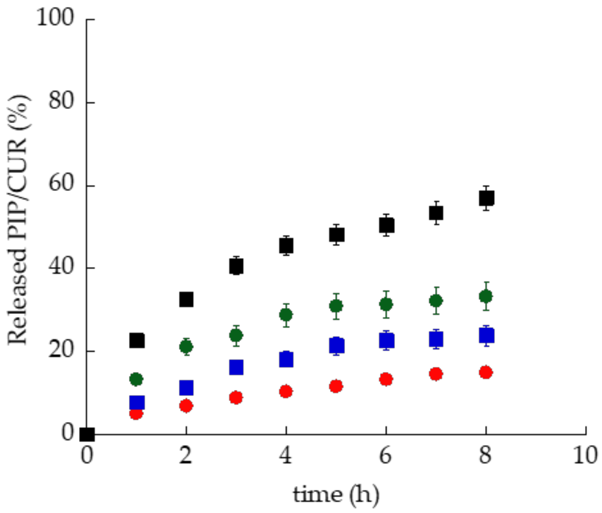

Figure S3. CUR and PIP release kinetics from ET-CUR (red circles), SOL-CUR (blue squares), ET-PIP (green circles) and SOL-PIP (black squares) as determined by Franz cells associated to PTFE membrane. The percentage refers to the amount of drug loaded in the formulations. Data are the mean of 6 independent experiments ± s.d.

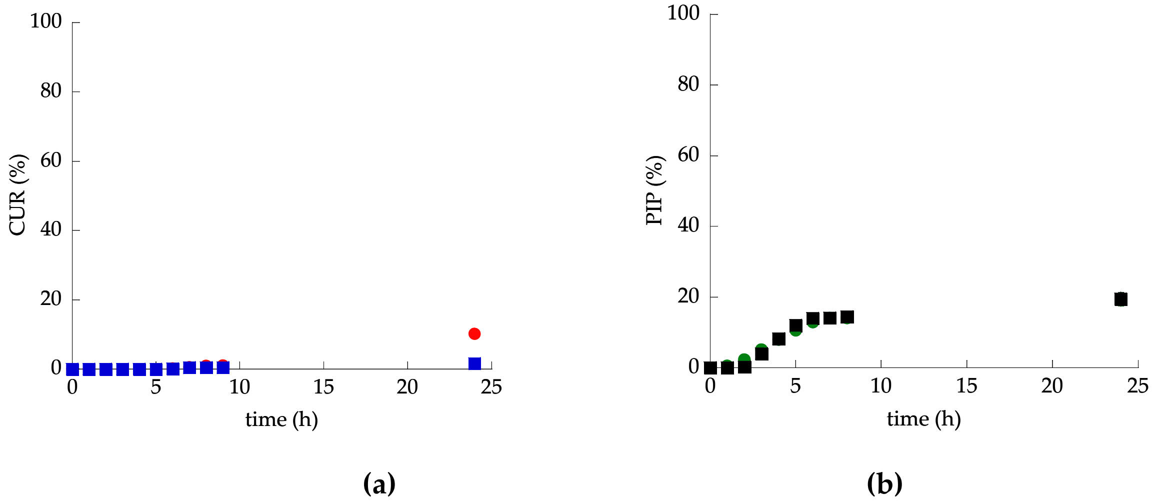

Figure S4. CUR **(a)** and PIP **(b)** diffusion kinetics from ET-CUR (red circles), SOL-CUR (blue squares), ET-PIP (green circles) and SOL-PIP (black squares) as determined by Franz cells associated to Strat-M membrane. Data are the mean of 6 independent experiments ± s.d. The percentage refers to the amount of drug loaded in the formulations. Data are the mean of 6 independent experiments ± s.d.
